# “Baking Powders” and the Public Health

**Published:** 1887-01

**Authors:** 


### "BAKING POWDERS" AND THE PUBLIC HEALTH.

The public have not the faintest conception, nor even the medical profession, an adequate idea of the extent to which "our daily bread" is adulterated; and, so cunningly is this adulteration effected in some instances, that not always do the "fittest" articles of food "survive." Many spurious, worthless, even dangerous preparations, by a kind of latter-day "booming," acquire a popularity which drives, often, purer, better articles from the market, on account of the lower price at which they can be sold. Instances of such adulteration are constantly being brought to light in those States where the Argus-eyed Inspector is ever on the alert. Recently, in New York, a large lot of "pure cider vinegar" (?) was seized, and on examination, was found to consist solely of water, acidulated with *elixir vitriol!* the cheapest commercial acid.

The adulteration of bread is more injurious to public health than any other form of food adulteration; and the sharpers "get in their work" most effectually through the medium of so-called "baking powders," with which the market is flooded,—all claiming to be the best, and *all* warranted "free from adulteration."

The substances most used for this nefarious purpose are alum and lime. Alum costs about 2½ cents per pound,—lime, almost nothing; while pure cream of tartar, of which these articles are claimed to be composed, costs about 40 cents: hence the temptation, and hence the successful imposition by means of lower price, upon the unsuspecting. Let the physician contemplate for a moment the effect which the daily ingestion of lime or alum must have, upon the general health. It is *a slow* poison, but a sure one, and is, doubtless, one of the most prolific causes of indigestion, dyspepsia, &c., and of kidney disease; to say nothing of a large class of nondescript ailments, which, being obscure, are laid to "malaria," that subtle essence, which, like the "thief in the community," has all sins laid at its door. Lime, upon being heated, gives off a small quantity of carbonic acid gas, which serves to make the bread "rise;" but what it is left?—quicklime,—a powerful caustic. Imagine the effect which the ingestion of such a substance daily, for a long period of time, must have upon the delicate mucous membrane of the stomach, especially of infants; and, later on, that of the bladder, where, doubtless, minute particles of it are finally deposited, and become the nuclei of the *stone in the bladder.*

Prof. J. W. Mallett, of the University of Virginia, (late of that of Texas), says, of this adulteration, under date of May 12, '86: "I have looked with some care, into the question of the admissibility of alum as an ingredient of baking powders to be used in preparing bread, and am decidedly of the opinion that both the substance (alum) itself, and the products to which it gives rise in the course of making of bread, are injurious to health, and on that account, fall into the list of such food accessories as should, on sanitary grounds, be prohibited by law."

The attention of the sanitary officers of the United States Government has been directed to this matter, and Prof. H. A. Mott, U. S. Gov't Chemist, made an analysis of over one hundred specimens of baking powders bought in open market, and it is asserted that every one that was examined, with *one* notable exception, the Royal Baking Powder, contained either alum or lime, or both; and in one, 12 per cent., or ⅛th of the weight, was lime. When such a conspicuous exception is found, amidst so much fraud, the public and the medical profession should show their appreciation of it; should regard it as a benefaction; and a part of the care exercised by the family physician on behalf of the health of his constituency, if directed to caution against such insidious adulterations, would, doubtless, prove a protective measure against future ailments, and save much subsequent trouble.

Actuated partly by curiosity, and partly by an interest in the subject, we caused an examination of this brand, the Royal

Baking Powder, to be made by a competent chemist, and assured ourselves of its purity. Doubtless there are other brands which are pure, also, or nearly so; but a conspicuous position has been given this one in particular, by published analyses, and testimonials of absolute purity by such men as Prof. H. A. Mott, Prof. G. W. Tucker, Professor of Chemistry in the Albany, N. Y., Medicai College; Prof. McMurtrie, late chemist of the Agricultural Departmeut of the U. S. Government; and by Prof. Mallett, of the Virginia University, well known to all Texans as a thorough and conscientious chemist.

It is not claimed that all baking powder adulterations are harmful, but it is to be urged that there is great danger in the use of those miscellaneous powders which flood the market, whose contents are not known, or are of doubtful quality. An illustration of this danger is given in the following paragraph, which is but one of the many similar reports seen in the public press:

WABASH, IND., Jan. 5.—The family of John Wooster, a prominent citizen of Montpelier, Blackford county, consisting of five persons, were poisoned on Monday night by eating biscuit in which had been used a very inferior quality of baking powder. Two hours after supper the entire family was seized with terrible gripes, and their intense pain not to be alleviated. The patients have grown steadily worse, and alarming symptoms have appeared. One of the children is dying, and the remainder of the family is in a precarious condition.—*Waco Examiner, Jan. 6.*

The hope is cherished, that the day is not far distant when an intelligent Board of Health in Texas will exercise a vigilance in the direction of food adulteration, in the interest of public health, and then this *crime*, for such it is, will be punished by law, as it deserves to be.

